# HIV-1 Virological Synapse is not Simply a Copycat of the Immunological Synapse

**DOI:** 10.3390/v2051239

**Published:** 2010-05-21

**Authors:** Gaia Vasiliver-Shamis, Michael L. Dustin, Catarina E. Hioe

**Affiliations:** 1 Program in Molecular Pathogenesis, Martin and Helen Kimmel Center for Biology and Medicine, Skirball Institute for Biomolecular Medicine, New York University School of Medicine, New York, NY 10016, USA; E-Mail: michael.dustin@nyumc.org; 2 Veterans Affairs New York Harbor Healthcare System, Manhattan Campus, Department of Pathology, New York University School of Medicine, New York, NY 10010, USA

**Keywords:** HIV-1, HIV, virological synapse, immunological synapse, HIV envelope, gp120, T cell receptor, CD4 T lymphocyte

## Abstract

The virological synapse (VS) is a tight adhesive junction between an HIV-infected cell and an uninfected target cell, across which virus can be efficiently transferred from cell to cell in the absence of cell-cell fusion. The VS has been postulated to resemble, in its morphology, the well-studied immunological synapse (IS). This review article discusses the structural similarities between IS and VS and the shared T cell receptor (TCR) signaling components that are found in the VS. However, the IS and the VS display distinct kinetics in disassembly and intracellular signaling events, possibly leading to different biological outcomes. Hence, HIV-1 exploits molecular components of IS and TCR signaling machinery to trigger unique changes in cellular morphology, migration, and activation that facilitate its transmission and cell-to-cell spread.

## 1. Introduction

Many viruses, including HIV-1, HTLV-1 and herpes viruses, disseminate to target cells much more effectively by cell-to-cell transmission as compared to cell-free virion transmission [1–5]. Cell-to-cell transmission has been observed to occur in an infectious or virological synapse (VS), where a tight cleft between an infected cell and a target cell is formed as a result of firmly adhering plasma membranes of the two apposing cells. The HIV-1 VS was first described for virus transmission from a dendritic cell to a T cell [6], but later on was also reported to form between infected T cells and target CD4 T cells [2]. Recently, formation of polysynapses between a single infected T cell and multiple target CD4 T cells has been described [7]. Such structures may be formed in secondary lymphoid tissues where a single infected cell may encounter many potential target cells simultaneously. Different types of HIV-1 VSs may be formed between virus-bearing dendritic cells or macrophages and target CD4 T cells [8–11] and between infected cells and epithelial cells [12–13]. However, little is currently known about the structural and molecular details of these VSs. In addition, other modes of HIV-1 cell-to-cell virus transmission involving nanotubes have also been observed [14], albeit less frequently [7].

This article will review the data on the HIV-1 VS formed between an infected CD4 T cell and a target CD4 T cell. The morphology of such VS has been postulated to mimic the immunological synapse (IS), which is formed when a T cell, via its TCR, recognizes the cognate peptide-MHC (pMHC) complex on an antigen-presenting cell (APC) [15]. IS formation is preceded by a stop signal that arrests cell migration and allows the two cells to interact and communicate efficiently in the busy cellular milieu [16]. This cell-cell communication subsequently leads to T cell activation and the induction of the T cell functions. Unlike IS, the VS formation does not necessarily involve TCR recognition of pMHC complexes; rather, its initial trigger is the binding of HIV-1 envelope glycoprotein gp120 (on the infected cell) to the target CD4 T cell [17–18]. The HIV-1 gp120 is non-covalently bound to gp41 to form heterotrimeric complexes on the surface of virions and infected cells. The gp120 subunit mediates virus interaction with the target cell by first binding to CD4 and then to the chemokine receptors (CKRs) CCR5 or CXCR4. The gp41 subunit contains the transmembrane anchor, the trimerization domain and the fusion peptide that enables virus fusion with the cell membrane and its entry into the target cell. After binding to CD4, gp120 undergoes conformational changes that enable it to bind the CKRs; this causes conformational changes in gp41, which expose the fusion peptide and thus initiate the fusion and entry of the virus into the target cell. Notably, HIV-1 VS formation is triggered by gp120 binding to CD4 and does not require the CKR engagement, although the gp120-CKR interactions are key for activating the gp41 subunit and for the subsequent fusion and entry processes [3,17–18]. In this article, we will discuss mainly the early cellular and biochemical events triggered by VS in the target CD4 T cells prior to virus entry. Recent reviews on virus assembly, trafficking, and budding in the infected cells are published elsewhere [19–21].

It is important to note that in addition to gp120 and its receptors, other membrane proteins, particularly the adhesion molecule LFA-1 and its ligands, the ICAM family, play a significant role in virus-cell interaction. These molecules are expressed on HIV-infected cells, target cells and the virus particles themselves [22–27]. Although these molecules are not absolutely required for HIV-1 infection, their presence significantly potentiates virus infectivity, cell-to-cell spread, and syncytium formation. ICAM-1 expression was often upregulated on primary T cells and lymphoid tissues infected with HIV-1 [27–28], leading to generation of HIV-1 virions with high levels of ICAM-1 on the envelope. Higher levels of ICAM-1 on virus particles correlated with enhanced virus infectivity and resistance to antibody-mediated neutralization [25–26, 29–30]. On the target cell side, LFA-1 expression promotes HIV-1 infection, replication and cell-to-cell transmission [23,31–32]. Regulation of LFA-1 activity through cytoskeleton remodeling and signaling components like PLCγ and ZAP70 also modulates the efficiency of HIV-1 entry into activated target T cell [32]. Notably, these adhesion molecules are found to be specifically recruited to and thus enriched within HIV-1 VS [2,33]. Since LFA-1-ICAM-1 interaction is known to be an integral component of the IS, the above observations lend support to the postulate that HIV-1 takes advantage of the structural apparatus of IS and forms unique synapses to enable its optimal cell-to-cell spread. In this review, we will highlight structural and functional similarities and differences between the HIV-1 VS and the IS, and how the specific features of the HIV-1 VS may be beneficial for virus dissemination.

## 2. Supramolecular structures of IS *versus* VS

Upon TCR engagement of a specific pMHC complex on an APC, a stop signal is triggered to arrest the highly migratory T cells onto a particular APC [16,34]. The migration arrest is followed by IS assembly, which is initiated by the formation of TCR/pMHC microclusters (MCs) in the periphery of T cell-APC contact area [35–38]. These MCs are then actively propelled in an actin-dependent manner to coalesce into the center of the contact area. Within 10 minutes, a mature IS is assembled and displays distinct areas known as supramolecular activation complexes (SMACs). The TCR/pMHC MCs coalescing at the center form the central SMAC (cSMAC), which is surrounded by a ring of LFA-1/ICAM-1 interactions known as the peripheral SMAC (pSMAC) (Figure 1A). The distal SMAC (dSMAC) surrounds the pSMAC and is enriched in CD45 and dynamic F-actin [39–41]. Other molecules also segregate into these SMACs; for examples, PKCθ clusters at the cSMAC in a CD28-dependent manner and talin is associated with the pSMAC through its interaction with LFA-1 [42–44]. As expected, CD4 can be found in the TCR/pMHC MCs and coalesces to the cSMAC, but soon thereafter it moves to the pSMAC [45]. It is also worth noting that CCR5 and CXCR4 are recruited to the IS interface, while CCR7 is not [46]. The exact mechanisms that regulate the segregation of various molecules into the distinct SMACs are not fully understood and remain a topic of active research. A current model for molecular segregation in the IS is size-dependent segregation [47]: the interacting TCR-pMHC molecules (spanning ∼13 nm) segregate from LFA-1-ICAM-1 interactions (spanning ∼30 nm) in order to optimize 2D affinity and active cytoskeletal transport move the distinct MCs into the respective SMACs [48]. By contrast, the distance spanned by the gp120-CD4 interaction can potentially reach up to 24–28 nm [49–50], which is similar to an extended LFA-1 molecule. Yet, as CD4 may partially lie along the membrane, the actual span of the gp120-CD4 complex may be closer in size to the TCR-pMHC interaction. Therefore, it is not immediately apparent that size differences alone would drive segregation of the CD4-gp120 complex from LFA-1-ICAM-1 [17,51]. A recent study further shows that the formation of the cSMAC also requires the action of TSG101 through recognition of ubiquitinated proteins [52], and interestingly this ESCRT-1 component also plays a key role in HIV-1 budding process [53–56].

Regardless of the mechanisms by which SMACs form, LFA-1 engagement of ICAM-1 is essential for IS formation both *in vitro* and *in vivo*. These molecules tether the opposing T cell and APC membranes within tens of nanometers, facilitating TCR-pMHC interactions. The symmetrical adhesion molecules ring, in particular, is essential for stopping locomotion while the dynamic actin-rich dSMAC is critical for sensitive antigen recognition [16,57–59]. The pSMAC also provides positional stability, which enhances T cell sensitivity to antigen as much as 100-fold [60]. Therefore, the molecular segregation in the IS is believed to allow T cells to integrate signaling efficiently [61]. Moreover, the IS allows directional transfer of signals or molecules between two distinct cells in the absence of cell-cell fusion [62]. The cSMAC is the delivery site of lytic granules in CD8 T cells [63] and cytokines in CD4 T cells [35] as well as the site for signaling termination, membrane recycling and TCR elimination [48,52].

Electron microscopy studies have revealed that budding virions and mature virus particles are concentrated in the synaptic cleft between HIV-infected cells and target CD4 T cells [7,64]. Budding viral crescents have also been also observed at the VS to protrude from the infected cell and directly contact the target cell membrane in the absence of mature virions [3]. The assembly and structural components of such VS have been studied in our laboratories using the planar bilayer system that mimics the infected cell membrane [17,51]. High-resolution microscopy of live CD4 T cells interacting with gp120 and ICAM-1 containing bilayers demonstrated that the VS is initiated by formation of gp120 MCs that coalesce to form a cSMAC-like structure [51], similar to the TCR MCs observed during IS assembly [36,37] (Table 1). The gp120 cSMAC is segregated from LFA-1-ICAM-1 interactions, which form an adhesive ring resembling the IS pSMAC (Figure 1B). This VS morphology is highly reminiscent of that of the mature IS (Figure 1A).

While the IS morphological structures have been confirmed by different labs studying T cell interactions with APCs or planar bilayers, the VS morphology and the exact localization of the different viral proteins in the VS SMAC structures remain controversial. The structural similarities of VS to IS in regard to LFA-1-ICAM-1 enrichment have been observed in cell-cell system [2,33] and confirmed with the planar bilayers system [17,51]. A recent study utilizing the cell-cell conjugate system further notes an accumulation of the abundant internal viral protein Gag into a button-like cluster in the VS center [3], reminiscent of the gp120 cSMAC. In contrast, Rudnicka *et al*. [7] have reported that Gag proteins may also aggregate in a ring-like structure, at least in a fraction of cell-cell conjugates analyzed. Yet, the precise localization and relative movement of HIV-1 Env as compared to Gag during VS formation remain unclear. Considerably lower levels of surface Env expression on infected cells and virus particles pose a technical obstacle for defining Env distribution with high resolution within the VS in the cell-cell conjugate system. The highly dynamic and transient nature of VS (described in the section below) also creates an additional challenge for capturing the full picture of molecular rearrangements taking place in such a synapse. Furthermore, HIV-1 VS is known to be enriched with other cellular proteins, such as ICAM-2 and ICAM-3 (both are also LFA-1 ligands) [33], tetraspanins [65], the lipid raft marker GM-1 [66] and the integrin α4β7 [67], but the temporal and spatial organization of these proteins during VS formation is not at all known. The biological importance of viral and cellular proteins at the HIV-1 VS needs to be better understood. Taking clues from its counterpart in the IS, the symmetrical LFA-1-ICAM-1 ring may mediate at least two functions: first, stopping cell migration and allowing fairly stable interactions between infected cells and target cells, and second, forming adhesive gasket that not only amasses virus particles produced by the infected cells into the synaptic cleft, but may also conceal the virus from the immune system during transfer to the target cell. Recent electron tomographic images of the HIV-1 VS demonstrate the presence of discrete adhesive regions that may be stabilized by integrins; however, this same study challenges the idea of the VS as a strategy for immune evasion since the VS appears to be permeable to anti-HIV antibodies and gp120-CKR blockers [64].

The initial formation of VS is induced by gp120-CD4 interaction, without involvement of CKRs. Therefore, only antibodies blocking gp120-CD4 binding prevent VS assembly, while antibodies and small antagonists interfering with gp120-CKR binding have no effect [17–18]. However, enrichment of specific CKRs at HIV VS has been demonstrated [2], although its precise distribution relative to other VS components is not known. Once the VS is formed, CKRs are required for the fusion process, but this can occur later after virus transfer across the synaptic cleft and even after VS is disassembled.

It is also evident that, unlike IS, VS can be formed in the absence of specific TCR-pMHC engagement (Table 1), although certain CD3 subunits have been shown to associate with VS and co-localize with gp120 in the VS cSMAC [2,51]. Weak recruitment of αβTCR and CD3ε was seen in the VS cSMAC and pSMAC, respectively, while the CD3ζ subunit was strongly phosphorylated and co-localized with gp120 in the VS cSMAC [51]. The mechanism for CD3ζ activation at VS in the absence of TCR-pMHC engagement is not fully understood. Yet, CD4-associated Lck was activated and localized to the VS cSMAC [51], thus Lck might phosphorylate components of the TCR-CD3 complex, which could be associated with CD4 at the VS. The biological contribution of CD3ζ activation to VS formation and virus infection are still unclear, although it is most likely responsible for the propagation of downstream signaling events observed at the VS [51] and will be discussed later. In the signaling cascade induced at the IS, activated Lck initiates signaling by phosphorylating the ITAMs on the CD3 chains and from there signaling propagates downstream (reviewed in [68]). Another intriguing question is how VS and IS may be formed when CD4 T cells encounter HIV-infected cells that also present the cognate pMHC complex. This is especially relevant as human CD4 T cells express MHC class II [69–70]. Arhel *et al*. [71] have shown that HIV infection does not disrupt IS formation between infected CD4 T cells and primary APCs and does not affect Lck recruitment to the IS. However, other studies claimed that HIV infection impairs IS formation, Lck recruitment and actin remodeling, and these effects were attributed to Nef [72–74]. Nevertheless, the type of synapse assembled between infected APCs and antigen-specific target CD4 T cells may be most pertinent for understanding HIV transmission and pathogenesis as virus-specific CD4 T cells and memory CD4 T cells in general have been shown to be the predominant cell populations targeted by the virus [75–78].

## 3. Structural stability of IS *versus* VS

Prior to antigenic exposure, the majority of T cells in the lymph nodes are highly motile, this motility is a key feature of T cells as it allows them to survey large areas in secondary lymphoid organs and increases the opportunity to encounter APCs. Upon TCR recognition of a specific pMHC on an APC, stable T cell-APC contact, which lasts for many hours, are established. These long-lived contacts have been observed *in vivo* using two-photon laser scanning microscopy under different experimental conditions with both CD4 and CD8 T cells (reviewed in [79]), and correspond with the formation of long-lasting mature IS seen when T cells interact with their cognate pMHC complexes presented on planar bilayers [17,80]. Indeed, the stable T cell-APC contacts have been shown to depend on LFA-1-ICAM-1 interactions [81], which create the adhesive pSMAC responsible for stopping T cell migration.

Although more transient T cell-APC contacts have been seen and are sufficient to initiate T cell activation, the long-lasting IS is essential for achieving full T cell differentiation and functions [79,81]. When the IS is maintained for many hours, T cells continue to receive activation signals, the integration of which may be responsible for determining differentiation pathways [82]. Comparative analyses of cytolytic CD4 and CD8 T cells also provided evidence that the stability of the cytolytic CD8 and CD4 IS correlated with their killing activity. The potently cytolytic CD8 T cells formed stable IS while the CD4 T cells, with weaker cytolytic activity, readily broke IS [83].

In comparison to IS, the HIV-1 VS formed on planar bilayers is relatively short-lived (Table 1). After VS is created by CD4 T cells on bilayers bearing HIV-1 gp120 and ICAM-1, the cells break the symmetrical LFA-1-ICAM-1 ring and resume migration. An average VS duration of 20–30 min was observed in the study [17]. By contrast, with the cell-cell system one study showed the mean life-time of the VS to be 60 min with a minimum duration of 10 min [64], while in another report the VS was observed to last between a few hours and up to 50 hours [3] (Table 1), although the cells were not studied under experimental conditions that allow cellular migration. When conjugates between uninfected CD4 T cells and HIV-1-infected monocyte-derived macrophages were evaluated, transient contacts were also noted as CD4 T cells detached soon after receiving the virus, but the contact duration was not quantified [11]. Nonetheless, HIV-1 antigen and receptor clustering, which marks the VS formation at the infected T cell-target T cell interface, was observed to peak within one hour and virus transfer was completed in three hours [2,18], indicating that fast and efficient virus transfer is not likely to require a long-lasting VS. Indeed, one may argue that the transient nature of VS would be beneficial for viral spread within lymphoid tissues; if the short VS duration is sufficient to allow virus transfer, the rapid detachment of the newly infected cells will promote their dispersion to other tissue sites. The newly infected T cells will subsequently express gp120, which in turn will arrest the migration of new target CD4 T cells, induce VS formation and continue virus propagation. The rate of virus spread can be further enhanced by the capacity of infected CD4 T cells to form VSs with multiple target cells either sequentially or simultaneously as recently reported [7].

The regulatory mechanisms that control VS dynamics and disassembly are yet to be determined. VS assembly and the preceding cell migration arrest are initiated within minutes upon gp120-CD4 interaction and without CKR involvement [17–18]. CKR recruitment has been shown to be relatively slow, with a lag phase of ∼30 min and a half-life of at least one hour [84], suggesting that CKR engagement at the VS is likely to occur after the virus is transferred to and docks onto CD4 on the target cells. At 30 min, the target CD4 T cells bearing the virus have started to detach and resume migration [17]. In the IS, the migration phase is driven by PKCθ [59], but PKCθ is not recruited to the VS (see below for further discussion). One potential trigger for VS disassembly may be the gp120-CKR interaction. HIV gp120 may mimic the chemokine ligands, as gp120 interaction with the CKR, independent of CD4, has been reported to cause chemotaxis that attracts or repels CD4 and CD8 T cells [85–87]. Consistent with this idea, CD4 T cell migration arrest induced by gp120, unlike the TCR-induced arrest, can be overcome by an SDF-1α chemokine gradient [17]. This suggests that T cell migration can resume when gp120 engages CKR. The relatively short VS duration and the delayed CKR involvement may also play a role in impeding syncytia formation, as syncytia are not a typical outcome of VS formation. The other cellular components specifically recruited to VS are likely to contribute, as well. For example, tetraspanins (CD9, CD63, CD81, and CD82) that are known to regulate cellular fusion are enriched at VS [65] and have been shown to influence HIV cell-cell transmission, but not cell-free virions infectivity [88–89]. Furthermore, high expression levels of tetraspanins were shown to suppress syncytium formation in a gag-dependent manner [89]. Lastly, the short duration of the VS implies that a complete T cell activation is not induced in the VS, as is in the IS, and this topic will be discussed in the following section.

## 4. T cell activation induced by IS *versus* VS

The formation of stable long-lived IS is crucial for generation of fully functional T cells at multiple stage of an immune response. Upon TCR engagement, a series of signaling events is triggered (reviewed in [68]), starting with activation of the Src kinases - Lck and Fyn, which in turn phosphorylate ITAMs on the CD3 chains (Figure 2). The phosphorylated ITAMs serve as a docking site for the Syk family kinase ZAP70, which phosphorylates LAT and SLP-76. LAT and SLP-76 are the basis for a larger multi-protein complex that propagates the signals downstream. The activation of SLP-76 promotes recruitment of Vav (a guanine nucleotide exchange factor), the adaptor proteins NCK, Grb2, and GADS, and an inducible T cell kinase (Itk). Itk subsequently phosphorylates phospholipase C γ1 (PLCγ1), which causes the hydrolysis of phosphatidylinositol 4,5-bisphosphate (PIP2) to produce the second messengers diacylglycerol (DAG) and inositol trisphosphate (IP3). DAG activates PKCθ and the MAPK/Erk pathways leading to transcription factor NF-κB activation. IP3 triggers Ca^2+^ influx and the Ca^2+^-associated calmodulin activates the phosphatase calcineurin to induce IL-2 gene transcription via the transcription factor NFAT.

The TCR-triggered signals are incorporated with signals to and from additional receptors, including LFA-1 (reviewed in [90]). For example, one outcome of TCR-induced signaling is LFA-1 activation via “inside-out” signaling. In addition, LFA-1 clustering after ligand binding also initiates “outside-in” signals that are transduced into cells via different kinases. These various activation signals are further linked to actin polymerization and other cytoskeleton rearrangements at the interface of the IS. The relationships between the cytoskeleton and signaling will be discussed later.

Considering the importance of long-lived IS, one surprising aspect of the spatiotemporal coordination of T cell activation is that in the mature IS, the cSMAC contains relatively low levels of active signaling [37]. Instead, active Lck, ZAP70, LAT, SLP-76, and Grb2 are strongly associated with the TCR MCs, which are generated in the periphery of the IS [36,38]. Another notable feature is that the TCR proximal signaling at an individual MC is remarkably brief, as the active signals are significantly reduced in two-to-three min during the time the MCs fuse into the cSMAC [37]. CD45, a phosphatase that downregulates Lck, was detected at higher levels in the cSMAC as compared to TCR MCs. Moreover, lyso-bis-phosphatidic acid (LBPA), a marker for multivesicular body (MVB) formation and degradation, was found at the cSMAC [37]. Taken together, this study suggests that the cSMAC is actually the termination site of TCR proximal signaling and TCR degradation. In a more recent study, it was shown that the cSMAC is divided into two structures: a TCR-rich center and an annular ring that contains CD28, its ligand CD80 and PKCθ [91]. This annular ring has been shown to be important for maintaining the co-stimulatory signals provided by CD28-CD80 interaction.

Since HIV-1 VS and IS share similar morphologies and many common structural components, one may postulate that T cell activation is one important biological consequence of the HIV-1 VS. HIV-1 replication in the host CD4 T cells is tightly tied to the T cell activation state, and thus the virus may exploit this activation mechanism to promote its replication. The activation of the target CD4 T cells, which newly acquire the virus via VS, is particularly of interest as it has a profound impact on the capacity of these newly infected cells to support virus replication. Completely quiescent CD4 T cells at G_0/1a_ phase are refractory to infection, due to the slow kinetics of the early steps of the virus replication including the inefficient reverse transcription [92], but suboptimal activation that moves the cells into the G1_b_ phase can be sufficient to render the cells susceptible to infection [93]. A number of studies have shown that gp120 can activate different signaling pathways [94–96] and trigger virus replication in HIV+ PBMCs without stimulating cellular proliferation and conventional activation markers [96–97]. R5 and X4 gp120 can induce the phosphorylation of CCR5 [98–99] and lead to Pyk2 activation [100]. R5 gp120 binding to CD4 on T cells also induces phosphorylation of Lck, ZAP70, Pyk2, paxilin and focal adhesion kinase (FAK) [98]. Even low concentrations of R5 and X4 envelope proteins and HIV-1 virions have been reported to induce Ca^2+^ influx in resting CD4 T cells [101]. Furthermore, gp120 interaction with resting CD4 T cells stimulates nuclear translocation of NFAT, which may promote virus infection in the cells that otherwise poorly support virus replication [94]. Lastly, gp120 has been shown to activate LFA-1 when engaging the α4β7 integrin [67]. By contrast, gp120 has been shown to cause caspase-3 and caspase-6 activation that can trigger pro-apoptotic signals and lead to cell death [102]. Further studies are needed to fully comprehend the regulatory balance that determines how and when gp120-induced signaling either benefit virus early replication events [95–97] or lead to target cell apoptosis [103–108].

Our recent study utilizing gp120 and ICAM-1 presenting planar bilayers and high resolution microscopy has evaluated TCR proximal signaling triggered in the target CD4 T cells in the VS [51]. The data showed that CD4 T cell interaction with gp120 and ICAM-1 induces phosphorylation of many components of the TCR signaling machinery including Lck, CD3ζ, ZAP70, LAT, SLP-76, Itk, and PLCγ. Yet, there was neither PKCθ recruitment nor induction of Ca^2+^ influx; the absence of these hallmarks of T cell activation indicates that the full T cell activation is not achieved in the VS. Figure 2 displays the activation of TCR proximal signaling detected at IS *versus* VS in the planar bilayer system. It is also important to note that ICAM-1 alone does not induce any TCR proximal signals, but gp120 alone is sufficient to activate some proximal TCR signals [109]. The contribution of gp120 binding to CD4 *versus* CKR is still unclear and needs further investigation.

Comparison of gp120-induced *versus* TCR-induced signals further demonstrates significant similarities and differences between the two synapses (Table 1). Lck-mediated signaling is initiated in gp120 MCs within five min, like that observed in TCR MCs, yet Lck activation lasts longer and is retained in the VS c-SMAC [51]. The VS cSMAC, unlike the IS cSMAC, is associated with activated signaling molecules. In fact, these active signals are still detected and found to co-localize with gp120-CD4 clusters after the VS disassembles and the cells resume migration. The spatiotemporal differences of the membrane proximal activation signals in the IS and VS are depicted diagrammatically in Figure 3. Nevertheless, further studies will need to determine whether the same signaling patterns are observed in VS formed in the cell-cell system. Virus-infected cells and virions themselves have a large array of other cell surface molecules, including MHC class I and class II, CD28, CD44, CD152, CD80, and CD86, all of which can interact with their ligands on the target CD4 T cells and influence positively or negatively the signaling induced in the VS. For example, when CD80 was added to the bilayer along with gp120 and ICAM-1, there was no change in the percentage of cells forming VS, but the levels of active Lck was increased and the levels of C-terminally phosphorylated Lck decreased [109]. Other potential players affecting activation signals via receptor cross-talks at VS are α4β7 [67] and CKRs beyond CCR5 and CXCR4 [110]. Moreover, additional studies are needed to discern the biologic significance of the durable signal transduction triggered in the CD4 T cells upon VS formation especially in terms of their effects on the CD4 T cells and on the virus. The current data provide evidence for the activation of proximal T cell signaling but not full T cell activation in the VS. Taken together, it is possible that HIV-1 VS instigates TCR proximal signaling events that partially activate the T cells to promote reverse transcription, integration or other steps in virus replication. At the same time these partial T cell activation signals may also cause aberrations in CD4 T cell functions, which are commonly linked with HIV infection.

## 5. Cytoskeleton dynamics in IS *versus* VS

IS assembly is tightly linked to the cytoskeleton dynamics. One component of the cytoskeleton is microtubules, which is pivotal for migration, directed vesicle secretion and structural support (reviewed in [111]). Within seconds after TCR stimulation, the microtubule-organizing center (MTOC) in the T cell polarizes to the IS interface [112–113]. This polarization is important for the trafficking and directed secretion of cytolytic granules and cytokines at the synapse [113–114]. At the VS, the MTOC polarizes toward the synaptic cell-cell contact area within the infected cell, but not within the target T cell. This pattern is consistently observed in HIV-1 VS and HTLV-1 VS [7,51,115–116]. In the target cell side, the absence of MTOC polarization is another indicator of the incomplete T cell activation induced in the VS. Quann *et al*. showed that localized DAG at the IS drives MTOC reorientation [117], but since PLCγ activation at the VS does not result in Ca^2+^ influx and PKCθ was also not recruited [51], there may not be enough DAG generated to drive MTOC polarization in the VS.

In addition to MTOC polarization, polymerization of filamentous (F)-actin marks a productive T cell activation [111]. The actin cytoskeleton also regulates the formation and stability of the IS. The TCR MCs formed at the IS periphery are associated with F-actin and the MC assembly and centripetal movement to the cSMAC are actin-dependent [36]. But once TCR MCs are formed, their stability is independent of actin. Interestingly, the cSMAC is relatively depleted of cortical actin compared to the dSMAC and pSMAC and an actin-polymerization inhibitor does not affect its integrity. The pSMAC, on the other hand, is enriched with F-actin (Figure 3), and this differential actin localization helps in shaping and maintaining the mature IS symmetry [63,118,119]. In addition, the actin cytoskeleton contributes to the maintenance of signaling complexes at the IS [120]; intact actin polymerization is critical for T cell signaling as its inhibition causes a rapid loss of Ca^2+^ influx [37].

In parallel to IS, the HIV-1 VS formation also involves actin polymerization. Inhibition of actin polymerization prevents conjugate formation between virus-infected cells and target cells [2] and blocks gp120 MCs formation observed in the planar bilayer experiments [51]. However, in contrast to the TCR MCs, gp120 MCs stability depends on actin (Table 1). Yet, just like in the IS cSMAC, an actin-depleted zone is created in the VS cSMAC (Figure 3) [51]. The differential pattern of actin polymerization observed at the VS may explain the paradoxical roles of actin in HIV-1 infection. Actin polymerization is induced by gp120-CD4 interaction and is required for CD4 and CKR recruitment [121–122]. In line with this finding, HIV-1 entry is inhibited when actin polymerization is blocked [123]. Once fusion and viral entry occurs, however, polymerized actin appears to pose a physical barrier for the virus, reducing virus infectivity at the post-entry stage [124,125]. The exact nature by which the actin inhibits infection still needs to be elucidated; but it does not seem to be a simple steric interference for the nuclear transport of the preintegration complex, as the spacing between the actin meshwork is larger than the size of the virus core [126]. One way the virus can overcome the actin barrier is by activating cofilin, an actin-severing factor. Gp120-CXCR4 interaction in resting T cells has been shown to activate cofilin and consequently facilitates viral nuclear integration and replication [125]. Nevertheless, for the most commonly circulating CCR5-tropic viruses, other pathways regulating the actin dynamics are likely involved and are yet to be discovered.

While the presence of an actin-depleted zone beneath the cSMAC is commonly noted in both VS and IS, the functions of this zone are unknown. Based on the functional consequences of actin dynamics on HIV entry and post-entry replication events discussed above, it can be hypothesized that the actin-free VS cSMAC can provide an ideal entry route for the virus. Therefore, HIV may have evolved to exploit to the VS by inducing partial T cell activation that results in actin clearance from the VS cSMAC. The underlying signaling events that trigger an actin-depleted cSMAC in both IS and VS are not fully understood. Lck signaling has been found to be necessary for the clearance of actin at the VS cSMAC [51], but the exact mechanism is not known. Further studies will also need to provide evidence for cofilin activation and recruitment in the VS, and for the signaling events linking Lck to cofilin. Of note, cofilin is known to play a role in F-actin dynamics at the periphery of IS [59], and may also contribute to the generation of actin-free zone in the cSMACs of IS [119] and VS. In addition, the ezrin, radixin, moesin (ERM) actin-binding proteins have been shown to be activated by HIV-1 gp120 and promote actin polymerization [121]. The activation of these cytoskeletal proteins facilitates the early steps of virus infection, but their involvement in the VS formation has not been evaluated. The ERM proteins are cleared from the IS interface to facilitate signaling [39]; hence, as Liu *et al*. suggested [126], it would be of interest to observe if the ERM proteins are activated and recruited to the VS. A better understanding of the similarities and differences between IS and VS may lead us toward new targets for prophylactic and therapeutic drugs against the virus.

A number of studies have reported that endocytosis may be one prominent route for HIV entry into its target cells [127–129] and this mode of virus entry was suggested to take place in the VS [3]. The lateral sorting of TCR into the cSMAC is mediated by endosomal sorting complexes required for transport (ESCRT) component TSG101 [52], which also plays a role in the viral budding machinery [53–56]. Furthermore, bidirectional membrane trafficking has been demonstrated in the activating natural killer cell synapse [130]. An intriguing question is whether the VS cSMAC, like its IS counterpart, is also enriched with proteins important for endocytosis, and if this structure is important for HIV-1 endocytosis. However, HIV-1 entry at the VS cSMAC, either via endocytosis or by direct fusion, is yet to be shown, and such data will provide the raison d’êtres for the HIV-1 VS.

## 6. Conclusions

HIV-1 utilizes common structural elements of the IS to form VS, but the assembled VS displays unique morphological features and dynamics. VS formation also induces partial T cell signaling events and cytoskeleton reorganization. Still, the current data about HIV-1 VS are very limited and more extensive studies are needed to further discern the roles of VS, not only in facilitating virus transmission, but also in inducing immunopathogenesis. Understanding the contributions of the HIV-1 VS in promoting viral spread and altering T cell immune functions will have a significant clinical impact. Ultimately this knowledge will be valuable for identifying novel preventive and therapeutic strategies for controlling HIV-1 infection and disease.

## Figures and Tables

**Figure 1.**
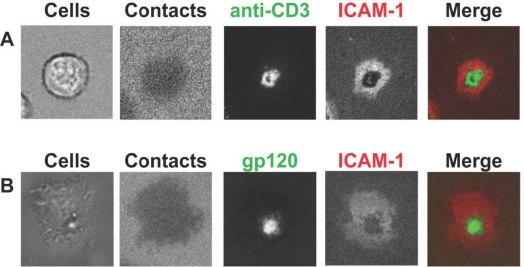
Morphological structures of IS *versus* HIV VS. **(a)** Mature IS is composed of a TCR-rich cSMAC and an adhesive pSMAC ring. **(b)** HIV VS displays a similar cSMAC and pSMAC segregation as observed in the IS, but the VS cSMAC is enriched with the virus envelope gp120.

**Figure 2.**
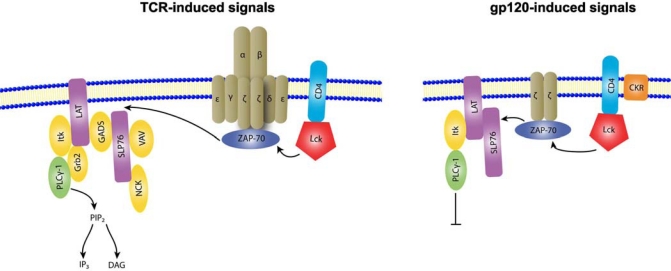
**Membrane proximal intracellular signals at the IS *versus* the HIV VS.** The interaction of TCR with its cognate pMHC complex triggers intracellular signaling events that lead to full T cell activation. The TCR proximal signals known to be activated at the IS are shown in the left diagram. Upon TCR engagement, Lck is activated and phosphorylates the TCR ITAMs. ZAP-70 is then recruited to the TCR ITAMs and phosphorylates LAT and SLP76. SLP76 subsequently recruits PLCγ-1 and other signaling molecules including Itk, Grb2, GADS, Nck and Vav. PLCγ-1 cleaves PIP_2_ to IP_3_ and DAG; IP3 triggers calcium influx while DAG activates PKCθ and the MAPK/Erk pathway. The signaling propagates downwards to activate transcriptions factors that alter gene transcription such as NF-κB and NFAT. The interaction of CD4 T cells with HIV-1 gp120 also triggers activation of some components of the TCR signaling machinery. The membrane proximal signals currently known to be activated at the VS in the planar bilayer experiments are shown in the right diagram.

**Figure 3.**
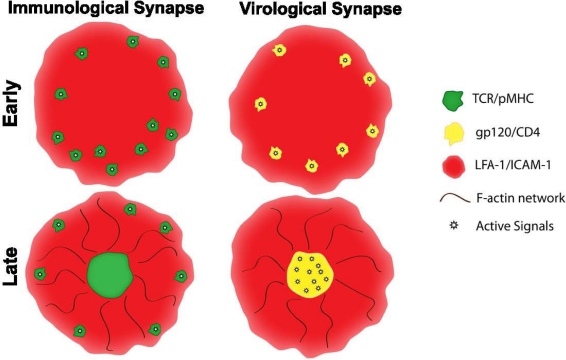
Diagrammatic illustrations of IS and VS at the early and later stage of the synapse assembly as observed in the planar bilayer system. The IS formation is initiated by TCR MCs that are associated with active signaling molecules such as Lck, ZAP70, LAT and SLP76 (top left). Within 2–3 minutes, TCR MCs converge to form the IS cSMAC, and active signaling is terminated. Active signaling, however, is sustained in the IS within the TCR MCs that are continually being formed at the periphery (bottom left). The VS is assembled from gp120 MCs that are also associated with active Lck (top right). However, in the VS, active signaling persists even after gp120 MCs converge to the cSMAC (bottom right). In both IS and VS, LFA-1-ICAM-1 interaction segregates to the pSMAC that surrounds the cSMAC, forming the hallmark bull’s eye structure. The actin cytoskeleton also rearranges to form an F-actin-depleted cSMAC in both synapses.

**Table 1.** Similarities and differences of IS and HIV VS.

	**Immunological Synapse**	**Virological Synapse**

Initial trigger	TCR-pMHC	gp120-CD4

Adhesion molecules involved	LFA-1-ICAM-1	LFA-1-ICAM-1,2 & 3

Synaptic structure		
Nascent	MCs: TCR and LFA-1 (LFA-1 essential)	MCs: GP120 and LFA-1 (LFA-1 optional)
Mature	cSMAC: TCR-pMHC	cSMAC: gp120-CD4
	pSMAC: LFA-1-ICAM-1	pSMAC LFA-1-ICAM-1
	dSMAC: CD45	dSMAC: ND
	MCs: TCR and LFA-1	MCs: ND

Synapse duration	Hours	20–30 minutes (planar bilayer system) 10 min to several hours (cell-cell system)

Signaling		
Initiation	TCR MCs (sustained in MCs)	gp120 MCs (sustained in cSMAC)
Termination	cSMAC	ND

Cytoskeleton:		
Actin	Required for MCs formation but not for stability cSMAC depleted of actin	Required for MCs formation and stability cSMAC depleted of actin
MTOC polarization	Yes	No: within the target T cell Yes: within the infected cell
Requirement for signaling	Intact actin required signaling	ND

ND, not determined

## References

[1] Igakura T, Stinchcombe JC, Goon PK, Taylor GP, Weber JN, Griffiths GM, Tanaka Y, Osame M, Bangham CR (2003). Spread of HTLV-I between lymphocytes by virus-induced polarization of the cytoskeleton. Science.

[2] Jolly C, Kashefi K, Hollinshead M, Sattentau QJ (2004). HIV-1 cell to cell transfer across an Env-induced, actin-dependent synapse. J Exp Med.

[3] Hubner W, McNerney GP, Chen P, Dale BM, Gordon RE, Chuang FY, Li XD, Asmuth DM, Huser T, Chen BK (2009). Quantitative 3D video microscopy of HIV transfer across T cell virological synapses. Science.

[4] Aubert M, Yoon M, Sloan DD, Spear PG, Jerome KR (2009). The virological synapse facilitates herpes simplex virus entry into T cells. J Virol.

[5] York IA, Johnson DC (1993). Direct contact with herpes simplex virus-infected cells results in inhibition of lymphokine-activated killer cells because of cell-to-cell spread of virus. J Infect Dis.

[6] Cameron PU, Freudenthal PS, Barker JM, Gezelter S, Inaba K, Steinman RM (1992). Dendritic cells exposed to human immunodeficiency virus type-1 transmit a vigorous cytopathic infection to CD4+ T cells. Science.

[7] Rudnicka D, Feldmann J, Porrot F, Wietgrefe S, Guadagnini S, Prevost MC, Estaquier J, Haase A, Sol-Foulon N, Schwartz O (2009). Simultaneous HIV Cell-to-Cell Transmission To Multiple Targets Through Polysynapses. J Virol.

[8] Arrighi JF, Pion M, Garcia E, Escola JM, van Kooyk Y, Geijtenbeek TB, Piguet V (2004). DC-SIGN-mediated infectious synapse formation enhances X4 HIV-1 transmission from dendritic cells to T cells. J Exp Med.

[9] Garcia E, Pion M, Pelchen-Matthews A, Collinson L, Arrighi JF, Blot G, Leuba F, Escola JM, Demaurex N, Marsh M, Piguet V (2005). HIV-1 trafficking to the dendritic cell-T-cell infectious synapse uses a pathway of tetraspanin sorting to the immunological synapse. Traffic.

[10] Yu HJ, Reuter MA, McDonald D (2008). HIV traffics through a specialized, surface-accessible intracellular compartment during trans-infection of T cells by mature dendritic cells. PLoS Pathog.

[11] Groot F, Welsch S, Sattentau QJ (2008). Efficient HIV-1 transmission from macrophages to T cells across transient virological synapses. Blood.

[12] Alfsen A, Yu H, Magerus-Chatinet A, Schmitt A, Bomsel M (2005). HIV-1-infected blood mononuclear cells form an integrin- and agrin-dependent viral synapse to induce efficient HIV-1 transcytosis across epithelial cell monolayer. Mol Biol Cell.

[13] Carreno MP, Krieff C, Irinopoulou T, Kazatchkine MD, Belec L (2002). Enhanced transcytosis of R5-tropic human immunodeficiency virus across tight monolayer of polarized human endometrial cells under pro-inflammatory conditions. Cytokine.

[14] Sowinski S, Jolly C, Berninghausen O, Purbhoo MA, Chauveau A, Kohler K, Oddos S, Eissmann P, Brodsky FM, Hopkins C, Onfelt B, Sattentau Q, Davis DM (2008). Membrane nanotubes physically connect T cells over long distances presenting a novel route for HIV-1 transmission. Nat Cell Biol.

[15] Piguet V, Sattentau Q (2004). Dangerous liaisons at the virological synapse. J Clin Invest.

[16] Dustin ML, Bromley SK, Kan Z, Peterson DA, Unanue ER (1997). Antigen receptor engagement delivers a stop signal to migrating T lymphocytes. Proc Natl Acad Sci USA.

[17] Vasiliver-Shamis G, Tuen M, Wu TW, Starr T, Cameron TO, Thomson R, Kaur G, Liu J, Visciano ML, Li H, Kumar R, Ansari R, Han DP, Cho MW, Dustin ML, Hioe CE (2008). Human immunodeficiency virus type 1 envelope gp120 induces a stop signal and virological synapse formation in noninfected CD4+ T cells. J Virol.

[18] Chen P, Hubner W, Spinelli MA, Chen BK (2007). Predominant mode of human immunodeficiency virus transfer between T cells is mediated by sustained Env-dependent neutralization-resistant virological synapses. J Virol.

[19] Campbell EM, Hope TJ (2008). Live cell imaging of the HIV-1 life cycle. Trends Microbiol.

[20] Bieniasz PD (2009). The cell biology of HIV-1 virion genesis. Cell Host Microbe.

[21] Chu H, Wang JJ, Spearman P (2009). Human immunodeficiency virus type-1 gag and host vesicular trafficking pathways. Curr Top Microbiol Immunol.

[22] Hioe CE, Bastiani L, Hildreth JE, Zolla-Pazner S (1998). Role of cellular adhesion molecules in HIV type 1 infection and their impact on virus neutralization. AIDS Res Hum Retroviruses.

[23] Hioe CE, Chien PC, Lu C, Springer TA, Wang XH, Bandres J, Tuen M (2001). LFA-1 expression on target cells promotes human immunodeficiency virus type 1 infection and transmission. J Virol.

[24] Bastiani L, Laal S, Kim M, Zolla-Pazner S (1997). Host cell-dependent alterations in envelope components of human immunodeficiency virus type 1 virions. J Virol.

[25] Fortin JF, Cantin R, Lamontagne G, Tremblay M (1997). Host-derived ICAM-1 glycoproteins incorporated on human immunodeficiency virus type 1 are biologically active and enhance viral infectivity. J Virol.

[26] Rizzuto CD, Sodroski JG (1997). Contribution of virion ICAM-1 to human immunodeficiency virus infectivity and sensitivity to neutralization. J Virol.

[27] Frank I, Stoiber H, Godar S, Stockinger H, Steindl F, Katinger HW, Dierich MP (1996). Acquisition of host cell-surface-derived molecules by HIV-1. Aids.

[28] Garrido M, Mozos A, Martinez A, Garcia F, Serafin A, Morente V, Caballero M, Gil C, Fumero E, Miro JM, Climent N, Gatell JM, Alos L (2007). HIV-1 upregulates intercellular adhesion molecule-1 gene expression in lymphoid tissue of patients with chronic HIV-1 infection. J Acquir Immune Defic Syndr.

[29] Paquette JS, Fortin JF, Blanchard L, Tremblay MJ (1998). Level of ICAM-1 surface expression on virus producer cells influences both the amount of virion-bound host ICAM-1 and human immunodeficiency virus type 1 infectivity. J Virol.

[30] Tardif MR, Tremblay MJ (2003). Presence of host ICAM-1 in human immunodeficiency virus type 1 virions increases productive infection of CD4+ T lymphocytes by favoring cytosolic delivery of viral material. J Virol.

[31] Groot F, Kuijpers TW, Berkhout B, de Jong EC (2006). Dendritic cell-mediated HIV-1 transmission to T cells of LAD-1 patients is impaired due to the defect in LFA-1. Retrovirology.

[32] Tardif MR, Tremblay MJ (2005). LFA-1 is a key determinant for preferential infection of memory CD4+ T cells by human immunodeficiency virus type 1. J Virol.

[33] Jolly C, Mitar I, Sattentau QJ (2007). Adhesion molecule interactions facilitate human immunodeficiency virus type 1-induced virological synapse formation between T cells. J Virol.

[34] Dustin ML (2009). Multiscale analysis of T cell activation: correlating *in vitro* and *in vivo* analysis of the immunological synapse. Curr Top Microbiol Immunol.

[35] Poo WJ, Conrad L, Janeway CA (1988). Receptor-directed focusing of lymphokine release by helper T cells. Nature.

[36] Campi G, Varma R, Dustin ML (2005). Actin and agonist MHC-peptide complex-dependent T cell receptor microclusters as scaffolds for signaling. J Exp Med.

[37] Varma R, Campi G, Yokosuka T, Saito T, Dustin ML (2006). T cell receptor-proximal signals are sustained in peripheral microclusters and terminated in the central supramolecular activation cluster. Immunity.

[38] Yokosuka T, Sakata-Sogawa K, Kobayashi W, Hiroshima M, Hashimoto-Tane A, Tokunaga M, Dustin ML, Saito T (2005). Newly generated T cell receptor microclusters initiate and sustain T cell activation by recruitment of Zap70 and SLP-76. Nat Immunol.

[39] Delon J, Kaibuchi K, Germain RN (2001). Exclusion of CD43 from the immunological synapse is mediated by phosphorylation-regulated relocation of the cytoskeletal adaptor moesin. Immunity.

[40] Allenspach EJ, Cullinan P, Tong J, Tang Q, Tesciuba AG, Cannon JL, Takahashi SM, Morgan R, Burkhardt JK, Sperling AI (2001). ERM-dependent movement of CD43 defines a novel protein complex distal to the immunological synapse. Immunity.

[41] Shaw AS, Dustin ML (1997). Making the T cell receptor go the distance: a topological view of T cell activation. Immunity.

[42] Huang J, Lo PF, Zal T, Gascoigne NR, Smith BA, Levin SD, Grey HM (2002). CD28 plays a critical role in the segregation of PKC theta within the immunologic synapse. Proc Natl Acad Sci USA.

[43] Monks CR, Freiberg BA, Kupfer H, Sciaky N, Kupfer A (1998). Three-dimensional segregation of supramolecular activation clusters in T cells. Nature.

[44] Freiberg BA, Kupfer H, Maslanik W, Delli J, Kappler J, Zaller DM, Kupfer A (2002). Staging and resetting T cell activation in SMACs. Nat Immunol.

[45] Kao H, Lin J, Littman DR, Shaw AS, Allen PM (2008). Regulated movement of CD4 in and out of the immunological synapse. J Immunol.

[46] Molon B, Gri G, Bettella M, Gomez-Mouton C, Lanzavecchia A, Martinez AC, Manes S, Viola A (2005). T cell costimulation by chemokine receptors. Nat Immunol.

[47] Springer TA (1990). Adhesion receptors of the immune system. Nature.

[48] Fooksman DR, Vardhana S, Vasiliver-Shamis G, Liese J, Blair D, Waite J, Sacristan C, Victora G, Zanin-Zhorov A, Dustin ML (2009). Functional Anatomy of T Cell Activation and Synapse Formation. Annu Rev Immunol.

[49] Liu J, Bartesaghi A, Borgnia MJ, Sapiro G, Subramaniam S (2008). Molecular architecture of native HIV-1 gp120 trimers. Nature.

[50] Wu H, Kwong PD, Hendrickson WA (1997). Dimeric association and segmental variability in the structure of human CD4. Nature.

[51] Vasiliver-Shamis G, Cho MW, Hioe CE, Dustin ML (2009). Human immunodeficiency virus type 1 envelope gp120-induced partial T-cell receptor signaling creates an F-actin-depleted zone in the virological synapse. J Virol.

[52] Vardhana S, Choudhuri K, Varma R, Dustin ML (2010). Essential role of ubiquitin and TSG101 protein in formation and function of the central supramolecular activation cluster. Immunity.

[53] Garrus JE, von Schwedler UK, Pornillos OW, Morham SG, Zavitz KH, Wang HE, Wettstein DA, Stray KM, Cote M, Rich RL, Myszka DG, Sundquist WI (2001). Tsg101 and the vacuolar protein sorting pathway are essential for HIV-1 budding. Cell.

[54] VerPlank L, Bouamr F, LaGrassa TJ, Agresta B, Kikonyogo A, Leis J, Carter CA (2001). Tsg101, a homologue of ubiquitin-conjugating (E2) enzymes, binds the L domain in HIV type 1 Pr55(Gag). Proc Natl Acad Sci USA.

[55] Martin-Serrano J, Zang T, Bieniasz PD (2001). HIV-1 and Ebola virus encode small peptide motifs that recruit Tsg101 to sites of particle assembly to facilitate egress. Nat Med.

[56] Perez OD, Nolan GP (2001). Resistance is futile: assimilation of cellular machinery by HIV-1. Immunity.

[57] Donnadieu E, Bismuth G, Trautmann A (1994). Antigen recognition by helper T cells elicits a sequence of distinct changes of their shape and intracellular calcium. Curr Biol.

[58] Negulescu PA, Krasieva TB, Khan A, Kerschbaum HH, Cahalan MD (1996). Polarity of T cell shape, motility, and sensitivity to antigen. Immunity.

[59] Sims TN, Soos TJ, Xenias HS, Dubin-Thaler B, Hofman JM, Waite JC, Cameron TO, Thomas VK, Varma R, Wiggins CH, Sheetz MP, Littman DR, Dustin ML (2007). Opposing effects of PKCtheta and WASp on symmetry breaking and relocation of the immunological synapse. Cell.

[60] Bachmann MF, McKall-Faienza K, Schmits R, Bouchard D, Beach J, Speiser DE, Mak TW, Ohashi PS (1997). Distinct roles for LFA-1 and CD28 during activation of naive T cells: adhesion *versus* costimulation. Immunity.

[61] Grakoui A, Bromley SK, Sumen C, Davis MM, Shaw AS, Allen PM, Dustin ML (1999). The immunological synapse: a molecular machine controlling T cell activation. Science.

[62] Dustin ML, Colman DR (2002). Neural and immunological synaptic relations. Science.

[63] Stinchcombe JC, Majorovits E, Bossi G, Fuller S, Griffiths GM (2006). Centrosome polarization delivers secretory granules to the immunological synapse. Nature.

[64] Martin N, Welsch S, Jolly C, Briggs JA, Vaux D, Sattentau QJ (2010). Virological synapse-mediated spread of human immunodeficiency virus type 1 between T cells is sensitive to entry inhibition. J Virol.

[65] Jolly C, Sattentau QJ (2007). Human immunodeficiency virus type 1 assembly, budding, and cell-cell spread in T cells take place in tetraspanin-enriched plasma membrane domains. J Virol.

[66] Jolly C, Sattentau QJ (2005). Human immunodeficiency virus type 1 virological synapse formation in T cells requires lipid raft integrity. J Virol.

[67] Arthos J, Cicala C, Martinelli E, Macleod K, Van Ryk D, Wei D, Xiao Z, Veenstra TD, Conrad TP, Lempicki RA, McLaughlin S, Pascuccio M, Gopaul R, McNally J, Cruz CC, Censoplano N, Chung E, Reitano KN, Kottilil S, Goode DJ, Fauci AS (2008). HIV-1 envelope protein binds to and signals through integrin alpha4beta7, the gut mucosal homing receptor for peripheral T cells. Nat Immunol.

[68] Smith-Garvin JE, Koretzky GA, Jordan MS (2009). T cell activation. Annu Rev Immunol.

[69] Costantino CM, Ploegh HL, Hafler DA (2009). Cathepsin S regulates class II MHC processing in human CD4+ HLA-DR+ T cells. J Immunol.

[70] Kaur G, Tuen M, Virland D, Cohen S, Mehra NK, Munz C, Abdelwahab S, Garzino-Demo A, Hioe CE (2007). Antigen stimulation induces HIV envelope gp120-specific CD4(+) T cells to secrete CCR5 ligands and suppress HIV infection. Virology.

[71] Arhel N, Lehmann M, Clauss K, Nienhaus GU, Piguet V, Kirchhoff F (2009). The inability to disrupt the immunological synapse between infected human T cells and APCs distinguishes HIV-1 from most other primate lentiviruses. J Clin Invest.

[72] Thoulouze MI, Sol-Foulon N, Blanchet F, Dautry-Varsat A, Schwartz O, Alcover A (2006). Human immunodeficiency virus type-1 infection impairs the formation of the immunological synapse. Immunity.

[73] Haller C, Rauch S, Fackler OT (2007). HIV-1 Nef employs two distinct mechanisms to modulate Lck subcellular localization and TCR induced actin remodeling. PLoS One.

[74] Rudolph JM, Eickel N, Haller C, Schindler M, Fackler OT (2009). Inhibition of T-cell receptor-induced actin remodeling and relocalization of Lck are evolutionarily conserved activities of lentiviral Nef proteins. J Virol.

[75] Douek DC, Brenchley JM, Betts MR, Ambrozak DR, Hill BJ, Okamoto Y, Casazza JP, Kuruppu J, Kunstman K, Wolinsky S, Grossman Z, Dybul M, Oxenius A, Price DA, Connors M, Koup RA (2002). HIV preferentially infects HIV-specific CD4+ T cells. Nature.

[76] Brenchley JM, Hill BJ, Ambrozak DR, Price DA, Guenaga FJ, Casazza JP, Kuruppu J, Yazdani J, Migueles SA, Connors M, Roederer M, Douek DC, Koup RA (2004). T-cell subsets that harbor human immunodeficiency virus (HIV) *in vivo*: implications for HIV pathogenesis. J Virol.

[77] Chomont N, El-Far M, Ancuta P, Trautmann L, Procopio FA, Yassine-Diab B, Boucher G, Boulassel MR, Ghattas G, Brenchley JM, Schacker TW, Hill BJ, Douek DC, Routy JP, Haddad EK, Sekaly RP (2009). HIV reservoir size and persistence are driven by T cell survival and homeostatic proliferation. Nat Med.

[78] Hermankova M, Siliciano JD, Zhou Y, Monie D, Chadwick K, Margolick JB, Quinn TC, Siliciano RF (2003). Analysis of human immunodeficiency virus type 1 gene expression in latently infected resting CD4+ T lymphocytes *in vivo*. J Virol.

[79] Bousso P (2008). T-cell activation by dendritic cells in the lymph node: lessons from the movies. Nat Rev Immunol.

[80] Dustin ML, Miller JM, Ranganath S, Vignali DA, Viner NJ, Nelson CA, Unanue ER (1996). TCR-mediated adhesion of T cell hybridomas to planar bilayers containing purified MHC class II/peptide complexes and receptor shedding during detachment. J Immunol.

[81] Scholer A, Hugues S, Boissonnas A, Fetler L, Amigorena S (2008). Intercellular adhesion molecule-1-dependent stable interactions between T cells and dendritic cells determine CD8+ T cell memory. Immunity.

[82] Yeh JH, Sidhu SS, Chan AC (2008). Regulation of a late phase of T cell polarity and effector functions by Crtam. Cell.

[83] Beal AM, Anikeeva N, Varma R, Cameron TO, Vasiliver-Shamis G, Norris PJ, Dustin ML, Sykulev Y (2009). Kinetics of early T cell receptor signaling regulate the pathway of lytic granule delivery to the secretory domain. Immunity.

[84] Gallo SA, Reeves JD, Garg H, Foley B, Doms RW, Blumenthal R (2006). Kinetic studies of HIV-1 and HIV-2 envelope glycoprotein-mediated fusion. Retrovirology.

[85] Brainard DM, Tharp WG, Granado E, Miller N, Trocha AK, Ren XH, Conrad B, Terwilliger EF, Wyatt R, Walker BD, Poznansky MC (2004). Migration of antigen-specific T cells away from CXCR4-binding human immunodeficiency virus type 1 gp120. J Virol.

[86] Iyengar S, Schwartz DH, Clements JE, Hildreth JE (2000). CD4-independent, CCR5-dependent simian immunodeficiency virus infection and chemotaxis of human cells. J Virol.

[87] Iyengar S, Schwartz DH, Hildreth JE (1999). T cell-tropic HIV gp120 mediates CD4 and CD8 cell chemotaxis through CXCR4 independent of CD4: implications for HIV pathogenesis. J Immunol.

[88] Krementsov DN, Weng J, Lambele M, Roy NH, Thali M (2009). Tetraspanins regulate cell-to-cell transmission of HIV-1. Retrovirology.

[89] Weng J, Krementsov DN, Khurana S, Roy NH, Thali M (2009). Formation of syncytia is repressed by tetraspanins in human immunodeficiency virus type 1-producing cells. J Virol.

[90] Mor A, Dustin ML, Philips MR (2007). Small GTPases and LFA-1 reciprocally modulate adhesion and signaling. Immunol Rev.

[91] Yokosuka T, Kobayashi W, Sakata-Sogawa K, Takamatsu M, Hashimoto-Tane A, Dustin ML, Tokunaga M, Saito T (2008). Spatiotemporal regulation of T cell costimulation by TCR-CD28 microclusters and protein kinase C theta translocation. Immunity.

[92] Zhou Y, Zhang H, Siliciano JD, Siliciano RF (2005). Kinetics of human immunodeficiency virus type 1 decay following entry into resting CD4+ T cells. J Virol.

[93] Korin YD, Zack JA (1998). Progression to the G1b phase of the cell cycle is required for completion of human immunodeficiency virus type 1 reverse transcription in T cells. J Virol.

[94] Cicala C, Arthos J, Censoplano N, Cruz C, Chung E, Martinelli E, Lempicki RA, Natarajan V, VanRyk D, Daucher M, Fauci AS (2006). HIV-1 gp120 induces NFAT nuclear translocation in resting CD4+ T-cells. Virology.

[95] Cicala C, Arthos J, Selig SM, Dennis G, Hosack DA, Van Ryk D, Spangler ML, Steenbeke TD, Khazanie P, Gupta N, Yang J, Daucher M, Lempicki RA, Fauci AS (2002). HIV envelope induces a cascade of cell signals in non-proliferating target cells that favor virus replication. Proc Natl Acad Sci USA.

[96] Misse D, Gajardo J, Oblet C, Religa A, Riquet N, Mathieu D, Yssel H, Veas F (2005). Soluble HIV-1 gp120 enhances HIV-1 replication in non-dividing CD4+ T cells, mediated via cell signaling and Tat cofactor overexpression. Aids.

[97] Kinter AL, Umscheid CA, Arthos J, Cicala C, Lin Y, Jackson R, Donoghue E, Ehler L, Adelsberger J, Rabin RL, Fauci AS (2003). HIV envelope induces virus expression from resting CD4+ T cells isolated from HIV-infected individuals in the absence of markers of cellular activation or apoptosis. J Immunol.

[98] Cicala C, Arthos J, Ruiz M, Vaccarezza M, Rubbert A, Riva A, Wildt K, Cohen O, Fauci AS (1999). Induction of phosphorylation and intracellular association of CC chemokine receptor 5 and focal adhesion kinase in primary human CD4+ T cells by macrophage-tropic HIV envelope. J Immunol.

[99] Su SB, Gong W, Grimm M, Utsunomiya I, Sargeant R, Oppenheim JJ, Ming Wang J (1999). Inhibition of tyrosine kinase activation blocks the down-regulation of CXC chemokine receptor 4 by HIV-1 gp120 in CD4+ T cells. J Immunol.

[100] Davis CB, Dikic I, Unutmaz D, Hill CM, Arthos J, Siani MA, Thompson DA, Schlessinger J, Littman DR (1997). Signal transduction due to HIV-1 envelope interactions with chemokine receptors CXCR4 or CCR5. J Exp Med.

[101] Melar M, Ott DE, Hope TJ (2007). Physiological levels of virion-associated human immunodeficiency virus type 1 envelope induce coreceptor-dependent calcium flux. J Virol.

[102] Cicala C, Arthos J, Rubbert A, Selig S, Wildt K, Cohen OJ, Fauci AS (2000). HIV-1 envelope induces activation of caspase-3 and cleavage of focal adhesion kinase in primary human CD4(+) T cells. Proc Natl Acad Sci USA.

[103] Arthos J, Cicala C, Selig SM, White AA, Ravindranath HM, Van Ryk D, Steenbeke TD, Machado E, Khazanie P, Hanback MS, Hanback DB, Rabin RL, Fauci AS (2002). The role of the CD4 receptor *versus* HIV coreceptors in envelope-mediated apoptosis in peripheral blood mononuclear cells. Virology.

[104] Biard-Piechaczyk M, Robert-Hebmann V, Richard V, Roland J, Hipskind RA, Devaux C (2000). Caspase-dependent apoptosis of cells expressing the chemokine receptor CXCR4 is induced by cell membrane-associated human immunodeficiency virus type 1 envelope glycoprotein (gp120). Virology.

[105] Roggero R, Robert-Hebmann V, Harrington S, Roland J, Vergne L, Jaleco S, Devaux C, Biard-Piechaczyk M (2001). Binding of human immunodeficiency virus type 1 gp120 to CXCR4 induces mitochondrial transmembrane depolarization and cytochrome c-mediated apoptosis independently of Fas signaling. J Virol.

[106] Herbeuval JP, Grivel JC, Boasso A, Hardy AW, Chougnet C, Dolan MJ, Yagita H, Lifson JD, Shearer GM (2005). CD4+ T-cell death induced by infectious and noninfectious HIV-1: role of type 1 interferon-dependent, TRAIL/DR5-mediated apoptosis. Blood.

[107] Lum JJ, Schnepple DJ, Badley AD (2005). Acquired T-cell sensitivity to TRAIL mediated killing during HIV infection is regulated by CXCR4-gp120 interactions. Aids.

[108] Trushin SA, Algeciras-Schimnich A, Vlahakis SR, Bren GD, Warren S, Schnepple DJ, Badley AD (2007). Glycoprotein 120 binding to CXCR4 causes p38-dependent primary T cell death that is facilitated by, but does not require cell-associated CD4. J Immunol.

[109] Vasiliver-Shamis G (2008).

[110] Edinger AL, Clements JE, Doms RW (1999). Chemokine and orphan receptors in HIV-2 and SIV tropism and pathogenesis. Virology.

[111] Billadeau DD, Nolz JC, Gomez TS (2007). Regulation of T-cell activation by the cytoskeleton. Nat Rev Immunol.

[112] Geiger B, Rosen D, Berke G (1982). Spatial relationships of microtubule-organizing centers and the contact area of cytotoxic T lymphocytes and target cells. J Cell Biol.

[113] Kupfer A, Dennert G, Singer SJ (1983). Polarization of the Golgi apparatus and the microtubule-organizing center within cloned natural killer cells bound to their targets. Proc Natl Acad Sci USA.

[114] Kupfer A, Mosmann TR, Kupfer H (1991). Polarized expression of cytokines in cell conjugates of helper T cells and splenic B cells. Proc Natl Acad Sci USA.

[115] Nejmeddine M, Negi VS, Mukherjee S, Tanaka Y, Orth K, Taylor GP, Bangham CR (2009). HTLV-1-Tax and ICAM-1 act on T-cell signal pathways to polarize the microtubule-organizing center at the virological synapse. Blood.

[116] Nejmeddine M, Barnard AL, Tanaka Y, Taylor GP, Bangham CR (2005). Human T-lymphotropic virus, type 1, tax protein triggers microtubule reorientation in the virological synapse. J Biol Chem.

[117] Quann EJ, Merino E, Furuta T, Huse M (2009). Localized diacylglycerol drives the polarization of the microtubule-organizing center in T cells. Nat Immunol.

[118] DeMond AL, Mossman KD, Starr T, Dustin ML, Groves JT (2008). T cell receptor microcluster transport through molecular mazes reveals mechanism of translocation. Biophys J.

[119] Kaizuka Y, Douglass AD, Varma R, Dustin ML, Vale RD (2007). Mechanisms for segregating T cell receptor and adhesion molecules during immunological synapse formation in Jurkat T cells. Proc Natl Acad Sci USA.

[120] Gomez TS, Billadeau DD (2008). T cell activation and the cytoskeleton: you can't have one without the other. Adv Immunol.

[121] Barrero-Villar M, Cabrero JR, Gordon-Alonso M, Barroso-Gonzalez J, Alvarez-Losada S, Munoz-Fernandez MA, Sanchez-Madrid F, Valenzuela-Fernandez A (2009). Moesin is required for HIV-1-induced CD4-CXCR4 interaction, F-actin redistribution, membrane fusion and viral infection in lymphocytes. J Cell Sci.

[122] Jimenez-Baranda S, Gomez-Mouton C, Rojas A, Martinez-Prats L, Mira E, Ana Lacalle R, Valencia A, Dimitrov DS, Viola A, Delgado R, Martinez AC, Manes S (2007). Filamin-A regulates actin-dependent clustering of HIV receptors. Nat Cell Biol.

[123] Iyengar S, Hildreth JE, Schwartz DH (1998). Actin-dependent receptor colocalization required for human immunodeficiency virus entry into host cells. J Virol.

[124] Campbell EM, Nunez R, Hope TJ (2004). Disruption of the actin cytoskeleton can complement the ability of Nef to enhance human immunodeficiency virus type 1 infectivity. J Virol.

[125] Yoder A, Yu D, Dong L, Iyer SR, Xu X, Kelly J, Liu J, Wang W, Vorster PJ, Agulto L, Stephany DA, Cooper JN, Marsh JW, Wu Y (2008). HIV envelope-CXCR4 signaling activates cofilin to overcome cortical actin restriction in resting CD4 T cells. Cell.

[126] Liu Y, Belkina NV, Shaw S (2009). HIV infection of T cells: actin-in and actin-out. Sci Signal.

[127] Miyauchi K, Kim Y, Latinovic O, Morozov V, Melikyan GB (2009). HIV enters cells via endocytosis and dynamin-dependent fusion with endosomes. Cell.

[128] Bosch B, Grigorov B, Senserrich J, Clotet B, Darlix JL, Muriaux D, Este JA (2008). A clathrin-dynamin-dependent endocytic pathway for the uptake of HIV-1 by direct T cell-T cell transmission. Antiviral Res.

[129] Daecke J, Fackler OT, Dittmar MT, Krausslich HG (2005). Involvement of clathrin-mediated endocytosis in human immunodeficiency virus type 1 entry. J Virol.

[130] Liu D, Bryceson YT, Meckel T, Vasiliver-Shamis G, Dustin ML, Long EO (2009). Integrin-dependent organization and bidirectional vesicular traffic at cytotoxic immune synapses. Immunity.

